# Impact of site, size and severity of ischemic cerebrovascular stroke on sleep in a sample of Egyptian patients a polysomnographic study

**DOI:** 10.1186/s12883-023-03438-6

**Published:** 2023-10-26

**Authors:** Jaidaa Mekky, Nadia Hafez, Osama El Kholy, Doaa Elsalamawy, Dina Gaber

**Affiliations:** Department of Neurology, Faculty of Medicine, Alexandria University, Alexandria, Egypt

**Keywords:** Stroke, Sleep, Polysomography

## Abstract

**Background:**

Sleep difficulties following a cerebrovascular stroke are an interesting topic in the scientific community. Following a brain ischemic insult, a variety of sleep problems can occur.

**Aim of work:**

To study the sleep architecture following stroke and to identify the impact of site, size and severity of ischemic cerebrovascular troke on sleep microstructure.

**Subjects and methods:**

this was a case control study; polysomnogram was done for 93 patients admitted to the stroke unit at El- Hadara university hospital with the first ever ischemic stroke. NIHSS was calculated immediately and 1 month after stroke onset. 50 age matched control subjects with no evidence of central nervous system or major psychiatric disorder by history or clinical examination.

**Results:**

Total sleep time, sleep efficiency were lower in ischemic cerebrovascular stroke patients than in control group and this was statistically significant (*p* = .001* *p* = .0001* respectively). Arousal index limb movement index and snoring index were all higher among the ischemic cerebrovascular stroke group in comparison to the control group and this was statistically significant (*p* = .0001*p = .05*p = .0001). Both the REM(rapid eye movement) and REM latency were highest among brain stem stroke, and this was statistically significant *p* = .043*, *p* = .0.001*.cortical infarcts showed higher AHI (apnea hypopnea index)and this was statistically significant *p* = 0.002* Limb movement index was higher among sizable size stroke and this was statistically significant (*p* = 0.038). NIHSS National Institutes of Health Stroke Scale after 1 month follow up showed a significant indirect correlation with the lowest oxygen saturation during sleep (*p* = 0.047). Lowest oxygen saturation was lowest among sizable stokes and desaturation index was highest among sizable size strokes both were statistically significant p = 0.006. NIHSS2 had a significant negative correlation with the lowest oxygen saturation during sleep *p* = 0.047.

**Conclusion:**

The microstructure of sleep is significantly impacted by cerebrovascular stroke. Brain stem strokes had the highest REM and REM latency, while cortical strokes had the highest moderate-to-severe AHI. Sizable strokes displayed increased indices of limb movement, desaturation, and oxygen saturation.

## Introduction

Numerous sleep disorders may manifest after a brain injury. Factors to consider may include the site of the brain lesion, the degree of the damage, variation in upper airway tone, the implications of comorbidities, and the type of treatment administered [1].

Prevalence of sleep-disordered breathing in stroke patients globally ranges from 30–70% of all stroke patients, as defined by an apnea -hypopnea index (AHI) > 15 /h [2].

Early detection and treatment strategies may help improve functional outcomes after a stroke because sleep disturbances may be associated with poorer stroke recovery and higher cerebrovascular morbidity [3].

In a metanalysis by liu X et al.,2021 obstructive sleep apnea (OSA)was shown to be a widely prevalent and manageable risk factor for ischemic cerebrovascular stroke [4].

Patients with sleep apnea who have had a stroke have longer rehabilitation and hospital stays, lower functional and cognitive results, and are more likely to experience further strokes [5].

Higher rates of endothelial dysfunction, oxidative stress, systemic inflammation, and the occurrence of atherosclerosis in stroke patients with sleep apnea may be attributed to intrathoracic pressure fluctuations, repeated arousals, and especially intermittent hypoxia [6].

Additionally, the persistent hypoxia and hypercapnia experienced by sleep apnea sufferers may impair cerebral circulation to the ischemic penumbra, aggravating brain damage [7].

However, in literature whether specific ischemic stroke locations or severity can cause sleep related breathing disorders is unknown.

Therefore, the aim in our study was to identify the impact of site size and severity of ischemic cerebrovascular stroke on sleep microstructure and hence help predict sleep related disorders after ischemic stroke.

### Participants and procedures

With 93 patients and 50 age-matched controls, this study was a case control study.

Inclusion criteria included 1)Patients with the first-ever ischemic stroke admitted to the stroke unit at El-Hadara university hospital (tertiary referral hospital, stroke unit, and sleep lab)0.2)Age range from 18 to 70 years 0.3) NIHSS ranging from 1 to 33. 4) good pulmonary and cardiac function.

Exclusion criteria included1) Previous haemorrhagic or cerebrovascular stroke, 2) any other unstable medical condition that would prevent participation (such as a deep coma, extreme obesity, or cardiac, renal, or hepatic failure),3) history of Cardiac or respiratory arrest within the past 3 months, 4)Myocardial infarction within the past 3 months,5) Severe pneumonia, 6)Previous pneumothorax, 7)Patients with COPD or Bullous emphysema, 8)Severe obesity (BMI above 30 were excluded) 9)ejection fraction below 50% 10)Children were excluded.

Ischemic cerebrovascular stroke was diagnosed clinically, Ct or MRI was performed for all patients, Echo was routinely done for all stroke patients. Pittsburgh Sleep Quality Index (PSQI) and The Epworth sleep scale before stroke (ESS1) were obtained from patients immediately post stroke and one month after stroke.

50 control volunteers without a history or clinical examination indicating a serious mental condition or a central nervous system disorder by using the same sleep questionnaire Pittsburgh Sleep Quality Index (PSQI) and The Epworth sleep scale before stroke (ESS1), little to no sleep disturbances were identified.

All patients had an overnight polysomnographic (PSG) testing within one week of the stroke's onset. The manufacturer's recommendations for the standard placement of the electrodes were followed. The sleep lab of the department of neurology hosted the PSG study utilising the data capture system Cadwell (Version 2.1, USA) [8] .

Two independent sleep specialists scored the PSG records according to the American Academy Standard Manual (AASM) for the scoring of sleep-associated events in version 2.6 (2022) [9].

### Data collection

Demographic and clinical data were collected from each patient and control, polysomnogram was done to all patients included in the study.Patients were divided in to 3groups according to the site of infarction; (A)subcortical,(B) cortical and (C) brain stem infarcts; their numbers were fifty-nine (63.44%), twenty four(25.81%), and ten (10.75%)respectively.

### Statistics and data analysis

All data were fed to a computer and analyzed using IBM Statistical Package for the Social Sciences (SPSS) software version 22.0. Qualitative variables were expressed as numbers and percentages. Quantitative data were expressed as mean or median, standard deviation, minimum, and maximum. The Kolmogorov- Smirnov test was utilized for verification of the normality of distribution of variables. Mann Whitney test was used to compare means between the studied groups, and Spearman's coefficient was used to study the correlation between EDSS scores and quantitative variables. Linear regression analysis was used to detect the most independent factors affecting EDSS scores. Significance of the obtained results was judged at the 5% level [10].

### Ethical approval and consent to participate

Ethical approval was obtained from the ethical committee (EC) of Alexandria University Faculty of Medicine which operates according to the International Conference of Harmonization Good Clinical Practice (ICH GCP) and applicable local and institutional regulations and guidelines [11]. The EC has a federal-wide assurance (FWA) [12] from 2010. Informed consents were obtained from the patients to use their anonymous data for research purposes. IRB NO 00012098, FWA NO 00018699. A written informed consent was obtained from all subjects prior to recruitment to the study. All methods were carried out in *accordance* with relevant guidelines and regulations.

### Data availability

The data are available upon request from the corresponding author.

## Results

### Demographic data

Patients age ranged from 24 to 84 years, with a mean of 55.05 ± 12.27 years while in the control group age ranged from 20 to 72 years with a mean of 51.9 ± 11.33 years.

Among the patients’ group 63.4% were males (59 patients) and 36.6% were females (34 patients).In the control group 29 were males (58.0%) and 21 females (42.0%) There was no statistical significance. (*p* = 0.524) between both groups.

### Sleep and stroke results

The score of the National Institute of Health Stroke Scale immediately following stroke ( NIHHS1) score ranged from 1 to 33,with a mean of 15.56 ± 7.703; while one month after stroke the NIHSS2 ranged from 1 to 31,with a mean of 10.59 ± 6.996. Comparing the patients NIHSS1 score to the NIHSS2 score of all patients immediately following the stroke and after one month period, it showed a very high significant improvement (*p* = 0.0001).

The Epworth sleep scale before stroke (ESS1) scores ranged from zero to 17 with a mean of 4.83 ± 3.778. Eight patients (8.6%) had an ESS score more than 9, which is the normal cutoff limit.One month later (ESS2) the mean was10.99 ± 4.569 and ranged from zero to 22, and 60 patients (64.5%) crossed the cutoff limit,i.e., the ESS 1 was worse one month following the stroke.The change was also very highly significant (*p* = 0 0.0001).

The Pittsburgh sleep scale before stroke ( PSS1) the score range was 0–18,and 1 month after (PSS2)it ranged from 0–19.The mean of the PSS1 was 5.56 ± 4.633,while the PSS 2 mean was 9.96 ± 5.267. Before the stroke the Pittsburgh sleep scale was lower(better) than the score one month after the stroke, and had a very high significance level (*p* = 0.0001) Table 1.

**Table 1:** Showing the NIHSS, PSS and ESS score change one month following the stroke

	**Scale**	**Mean**	**Std. Deviation**	**t**	
**Pair 1**	NIHSS 1	15.56	7.703	11.752	.0001*
	NIHSS 2	10.59	6.996		
**Pair 2**	PSS1	5.56	4.633	10.372	.0001*
	PSS 2	9.96	5.267		
**Pair 3**	ESS1	4.83	3.778	12.806	.0001*
	ESS2	10.99	4.569		

*NIHSS1* National Institute of Health Stroke Scale immediately following stroke, *NIIHSS2* National Institute of Health Stroke Scale 1 month after stroke, *ESS1* Epworth sleep scale before stroke, *ESS2* Epworth sleep scale 1 month after stroke, *PSS1* Pittsburgh sleep scale before stroke, *PSS2* Pittsburgh sleep scale 1 month after stroke

*•P* > 0.05 = Non Significant, *P* ≤ 0.05 = Significant

*•P* ≤ 0.01 = Highly significant, *P* ≤ 0.005 = Very highly significant

### Polysomnography results

#### Patients versus control

The total sleep time (TST) and sleep efficiency (SE) were higher in the control group than in the studied patients, this was statistically significant (*p* = 0.001).

While sleep latency and Stage changes per hour of sleep (S.CH) were higher in patients than in control group.

Regarding sleep architecture, slow wave sleep was higher in stroke patients than in the control group while REM and REM latency were higher in the control group than in stoke patients and this was statistically significant.

The arousal index (AI) limb movement index (LMI) snoring index (SI) were higher in stroke patients than in the control group and this difference was statistically significant.

The apnea hypopnea index (AHI), Obstructive sleep apnea index (OSA) central sleep apnea (CSA), hypopnea index (HYP) as well as the desaturation index (DI), were all higher in the stroke patients’ group than in the control group and this was statistically significant.Contrary to Mixed apneas (MA) which was higher among the control group.

Lowest oxygen saturation during sleep (LO2) was recorded among the stroke patients Table 2.

#### Stroke site (Cortical, Subcortical, Brain stem)

**Table 2:** Comparison between patients and control regarding sleep respiratory study

	**Group**	**Min**	**Max**	**Mean**	**S.D**	**F**	**Sig**
**AHI**	Patients	0	59	14.17	14.478	13.237	.0001*
	Control	0	21	5.97	9.061		
**OSA**	Patients	0	40	7.20	9.872	15.355	.0001*
	Control	0	18	1.59	3.042		
**CSA**	Patients	0	30	1.77	3.933	8.535	.004*
	Control	0	1	.14	.273		
**M A**	Patients	0	30	1.12	3.324	4.350	.039*
	Control	0	12	2.32	3.236		
**HYP**	Patients	0	44	3.73	5.811	18.141	.0001*
	Control	0	2	.22	.473		
**LO2**	Patients	40	95	76.55	15.805	15.840	.0001*
	Control	56	98	86.30	9.590		
**DI**	Patients	.1	85.0	19.516	18.7842	41.157	.0001*
	Control	.0	33.0	2.136	4.9256		

*AHI* Apnea hypo apnea index, *OSA* Obstructive sleep apnea, *CSA* Central sleep apnea, *MA* Mixed apnea, *HYP* Hypo apnea index, *LO2* Lowest oxygen saturation, *DI* Desaturation index

The stroke subtypes, whether cortical, subcortical or brainstem differed in the REM % and REM latency, both the REM and REM latency were highest among brain stem stroke, and this was statistically significant (Table 3).

**Table 3:** Comparison between patient’s subgroups regarding sleep architecture

Stage%	Group	Min	Max	Mean	S.D	F	Sig
**Stage 1%**	A	1	28	7.59	5.143	1.327	.270
	B	2	13	6.63	3.019		
	C	0	12	5.20	4.077		
**Stage 2%**	A	33	76	53.75	10.856	1.159	.319
	B	26	72	50.96	10.813		
	C	41	57	49.30	4.900		
**Stage3&4%**	A	2	45	22.03	9.759	.905	.408
	B	10	42	21.58	8.070		
	C	18	43	26.00	8.138		
**REM%**	A	0	50	16.44	9.293	3.248	.043*
	B	0	60	21.17	11.343		
	C	14	29	22.60	4.088		
**REM L**	A	25	180	72.6	27.1	8.01	0.001*
	B	25	181	79.49	33.589		
	C	59	175	99.11	27.589		

*A* Subcortical infarcts, Cortical infarcts, *C* Brain stem infarcts

*1%* Percentage of stage 1, *2%* Percentage of stage 2, *3&4%* Percentage of stage 3& 4, *REM %* Percentage of stage *REM* REM L REM latency)

*•P* > 0.05 = Non Significant, *P* ≤ 0.05 = Significant

*•P* ≤ 0.01 = Highly significant, *P* ≤ 0.005 = Very highly significant

There was no statistical difference between, cortical, subcortical and brainstem infarcts as regard the total sleep time (*p* = 0.739), sleep latency (*p* = 0.084), sleep efficiency (*p* = 0.793) and stage changes per hour of sleep (*p* = 0.418) (Table 4).

**Table 4:** Relation between patients’ subgroups regarding sleep parameters

	**Group**	**Min**	**Max**	**Mean**	**S.D**	**F**	**Sig**
**TST**	A	39	601	348.94	124.248	.304	.739
	B	57	520	332.25	113.936		
	C	193	477	323.30	95.567		
**SL**	A	0	65	13.41	11.590	2.545	.084
	B	0	51	18.73	12.764		
	C	3	29	10.25	7.330		
**S.E**	A	1	98	80.29	15.349	.232	.793
	B	8	95	78.00	18.249		
	C	58	95	81.40	12.730		
**S.CH**	A	18	395	105.95	48.338	.881	.418
	B	23	134	93.17	25.698		
	C	65	132	107.40	20.217		

*A* Subcortical infarcts, Cortical infarcts, *C* Brain stem infarcts

*TST* Total sleep time, *SL* Sleep Latency, *SE* Sleep efficiency, *S.CH* Stage Changes per hour of Sleep

*•P* > 0.05 = Non Significant, *P* ≤ 0.05 = Significant

*•P* ≤ 0.01 = Highly significant, *P* ≤ 0.005 = Very highly significant

Regarding stroke localization and AHI grades apnea was highest among the cortical infarction group, severe apnea was present among cortical subcortical and brainstem infarcts in a near range while no apnea was highest among brain stem infarcts (Table 5).

#### Stroke Size (lacunar versus sizable Strokes)

**Table 5:** Comparison between patients’ subgroups regarding AHI score

AHI score	Total							
	**A**		**B**		**C**		**Total**	
	No	%	No	%	No	%	No	%
**No**	18	30.5%	7	29.2%	6	60.0%	64	44.8%
**Mild**	14	23.7%	7	29.2%	1	10.0%	29	20.3%
**Moderate**	8	13.6%	1	4.2%	0	0%	16	11.2%
**Severe**	19	32.2%	9	37.5%	3	30.0%	34	23.8%
Total	59	100.0%	24	100.0%	10	100.0%	143	100.0%
X^2^ P	25.61 0.002*							

*AHI* Apnea Hypopnea index

*A* Subcortical infarcts *B* Cortical infarcts, *C* Brain stem infarcts

*•P* > 0.05 = Non Significant, *P* ≤ 0.05 = Significant

*•P* ≤ 0.01 = Highly significant, *P* ≤ 0.005 = Very highly significant

Thirty-five patients (37.63%) had a sizable infarct, while the rest -fifty-eight (62.36%) had a lacunar infarct. The limb movement index was higher among sizable strokes and this was statistically significant p value of 0.038 (Table 6).

**Table 6:** Relation between the stroke size regarding sleep indices

	**Size**	**N**	**Mean**	**Std. Deviation**	**Min**	**Max**	**t**	**Sig**
**AI**	lacunar	58	17.12	12.328	0	59	2.313	.132
	sizable	35	22.41	21.266	3	98		
**LMI**	lacunar	58	2.37	3.282	0	13	4.441	.038*
	sizable	35	4.52	6.533	0	22		
**SI**	lacunar	58	15.209	13.8744	.3	62.0	1.175	.281
	sizable	35	18.269	11.9620	.7	43.0		

*AI* Arousal index, *LMI* Limb movement index, *SI* Smoking index

*•P* > 0.05 = Non-Significant, *P* ≤ 0.05 = Significant

*•P* ≤ 0.01 = Highly significant, *P* ≤ 0.005 = Very highly significant

The lowest oxygen saturation was lowest among sizable stoke and desaturation index was highest among sizable size stroke both were statistically significant (Table 7).

#### Stroke severity

**Table 7:** Relation between the stroke sizes regarding sleep respiratory study

	**Size**	**N**	**Mean**	**Std. Deviation**	**Min**	**Max**	**t**	**Sig**
**AHI**	lacunar	58	12.47	14.654	0	59	2.149	.146
	Sizable	35	16.99	13.932	0	48		
**OSA**	lacunar	58	5.80	8.938	0	39	3.211	.076
	Sizable	35	9.54	10.990	0	40		
**CSA**	lacunar	58	1.40	4.157	0	30	1.340	.250
	Sizable	35	2.37	3.505	0	16		
**MA**	lacunar	58	1.33	4.143	0	30	.622	.432
	Sizable	35	.77	.962	0	4		
**HYPO**	lacunar	58	3.77	6.615	0	44	.008	.928
	Sizable	35	3.66	4.242	0	20		
**L O2**	lacunar	58	80.01	14.893	1	95	7.895	.006*
	Sizable	35	70.84	15.816	0	91		
**DI**	lacunar	58	15.576	19.3683	.1	85.0	7.241	.008*
	Sizable	35	26.046	15.9841	5.2	81.0		

*AHI* Apnea Hypopnea index, *OSA* Obstructive sleep apnea, *CSA* Central sleep apnea, *MA* Mixed sleep apnea, *HYP* Hypopnea, *L0 2* Lowest oxygen saturation during sleep, *DI* Desaturation index

*•P* > 0.05 = Non Significant, *P* ≤ 0.05 = Significant

*•P* ≤ 0.01 = Highly significant, *P* ≤ 0.005 = Very highly significant

The sleep architecture was not affected by the stroke severity, nor was the REM latency.

(Table 8).

**Table 8:** Relation between NIHSS and sleep architecture

		**NIHSS 1**	**NIHSS 2**
**1%**	Pearson Correlation	.001	-.046
	Sig. (2-tailed)	.989	.663
**2%**	Pearson Correlation	.102	.083
	Sig. (2-tailed)	.329	.427
**3 & 4%**	Pearson Correlation	-.171	-.120
	Sig. (2-tailed)	.102	.251
**REM%**	Pearson Correlation	.051	.046
	Sig. (2-tailed)	.627	.661
**REM L**	Pearson Correlation	.055	.201
	Sig. (2-tailed)	.768	.279

*1%* Percentage of stage 1, *2%* Percentage of stage 2, *3&4%* Percentage of stage 3& 4,

*REM %* Percentage of stage REM, *REM L* REM latency

*•P* > 0.05 = Non Significant, *P* ≤ 0.05 = Significant

*•P* ≤ 0.01 = Highly significant, *P* ≤ 0.005 = Very highly significant

NIHSS was calculated twice immediately, NIHSS1and after 1-month NIHSS2.

The stroke severity, NIHSS2, had a significant indirect correlation with the lowest oxygen saturation during sleep. The p value was 0.047 (Table 9).

**Table 9:** Relation between NIHSS and sleep respiratory study

		**NIHSS 1**	**NIHSS 2**
**AHI**	Pearson Correlation	.059	.032
	Sig. (2-tailed)	.574	.758
**OSA**	Pearson Correlation	.033	.097
	Sig. (2-tailed)	.754	.357
**CSA**	Pearson Correlation	.125	.002
	Sig. (2-tailed)	.234	.985
**MA**	Pearson Correlation	-.030	-.099
	Sig. (2-tailed)	.776	.346
**HYPO**	Pearson Correlation	-.003	.043
	Sig. (2-tailed)	.980	.685
**L O2**	Pearson Correlation	-.154	-.207
	Sig. (2-tailed)	.141	.047 *
**DI**	Pearson Correlation	.164	.146
	Sig. (2-tailed)	.116	.161

*AHI* Apnea Hypopnea index, *OSA* Obstructive sleep apnea, *CSA* Central sleep apnea, *MA* Mixed sleep apnea, *HYP* Hypopnea, *L0 2* Lowest oxygen saturation during sleep, *DI* Desaturation index

*•P* > 0.05 = Non Significant, *P* ≤ 0.05 = Significant

*•P* ≤ 0.01 = Highly significant, *P* ≤ 0.005 = Very highly significant

## Discussion

In literature the impact of stroke on sleep microstructure have been described yet the impact of stroke site,size and location showed variable and sometimes contradicting results in our study we aimed to study the effect of these variables.

### Patients versus control

Several studies showed markedly disturbed sleep microstructure after stroke including a pilot study by *Hofmeijer *et al. [13] and a prospective study by *Šiarnik *et al. [14] they observed significantly higher values of apnea–hypopnea index (24.8 vs. 7.6, *p* = 0.007), desaturation index ([DI] 26.9 vs. 8.8, *p* = 0.005), arousal index (22.6 vs. 13.1, *p* = 0.035). These results matched our study, which emphasized the fact that the overall sleep quality and sleep continuity are disturbed in post stroke patients.

*Terzoudi *et al. [15] showed that stroke patients (without sleep disordered breathing) had reductions in total sleep time and sleep efficiency matching our study but a reduced stage II and slow wave sleep and increased sleep latency. This was showed in 2019 in a study by Julio Fernandez-Mendoz et al. which showed that the likelihood of death in middle-aged persons with cardiometabolic risk factors was predicted by short sleep duration [16].

The limb movement index was significantly higher among ischemic stroke patients, which were also the results by *Sechi *et al. [17] *Kang *et al. [18].

*Enduri *et al. [19] studied a total of 128 patients with stroke or TIA and 62.5% had a high risk of sleep apnea, and only three of these had a previous diagnosis of obstructive sleep apnea, while *Lipford *et al. [20] showed strong association between obstructive sleep apnea (OSA) and cardio embolic (CE) stroke. this was further highlighted in a study by Woo-Jin Lee et al. in 2021, which showed that obstructive sleep apnea is linked to both lacunar vessel disease and decreased cerebrovascular compliance and suggested that there might be a region-specific underlying pathognomonic relationship [21].

### The stroke site

The relationship between obstructive sleep apnea (OSA) and stroke location is conflicting with some studies finding an association and others demonstrating no relationship. In our study moderate to severe AHI was present among cortical strokes. *Stahl *et al. study indicated that OSA is present in most stroke patients and imply that stroke location cannot be used to identify a group with higher risk of OSA. Their results also suggest that OSA likely predated the stroke [22].

This was also concluded form a study done by *Mansour *et al. in Egypt which stated that there was no correlation between the occurrence of sleep disorders and the site of the stroke [23].

*Hermann *et al. stated that patients with thalamic stroke revealed a non-significantly lower sleep efficiency, a significantly higher proportion of stage 1, and a significantly lower proportion of stage 2 sleep. Stages 3/4 and rapid eye movement sleep were not significantly altered. Sleep spindles were decreased in patients with paramedian thalamic stroke when compared with control subjects. These results were related to thalamic infarcts only, but not to all subcortical strokes [24].

Similar results were found by *Harbison *et al., who said that the mean AHI was higher in subjects with lacunar vs. cortical strokes. Pre-stroke sleepiness was associated with post-stroke neurological impairment and disability.In this study the AHI was higher in subcortical infarcts, but this difference did not reach the level of significance. This difference may be due to the different time interval chosen for the sleep study following the stroke [25].

In a prospective observational study done by *Fisse *et al. which included 142 sub-acute stroke patients,MRI was performed to all patients sleep disordered breathing group showed a higher NIHSS, yet the prevalence and clinical characteristics of sleep disordered breathing did not show significant differences among stroke types or locations [26].

Contrary to our results Brown et al. performed a Cross-sectional study on 38 brain stem stroke patients and found that acute infarction involving the brainstem was associated with both presence and severity of sleep disordered breathing. The difference might be due to the smaller sized stroke in brainstem in our study to allow Polysomnography to be done safely for patients [27].

Gupta et al. studied 346 stroke patients 10.11% had RLS they also concluded that RLS, especially unilateral or asymmetrical, may frequently pre-exist in patients presenting with subcortical stroke [28].

### The stroke size

Stroke size showed significance affecting the limb movement index, which denotes that the larger the damage to the basal ganglia and motor circuit, the higher the periodic limb movements.

The lowest oxygen saturation was highly significant, as it was lower in cases with sizable infarcts. Desaturation index was higher in sizable infarcts. This supports the theory that frequent desaturation and the intermittent hypoxia have a long term negative effect on the cerebral hemodynamics and do contribute in worse stroke outcome.

In the research of Basetti and Aldrich [29] patients with acute hemispheric stroke had less total sleep time lower sleep efficiency, less NREM stage 2 sleep and less non rapid eye movement (NREM) stage 3,4 sleep. No significant differences were found between stroke patients and controls in REM sleep measures. The difference in results may be attributed to the sizable extent of the infarct. Also, the study conducted by Basetti was performed in the acute phase ( first three days) of stroke and usually the marked changes in sleep following stroke tend to ameliorate with time.

Different from the current results were those found in a study conducted by Gottselig, where he conducted his study on 72 patients with hemispheric stroke,where the durations of rapid eye movement and non-rapid eye movement sleep stages did not differ significantly between brain-damaged subjects and hospitalized controls. On the other hand they showed significantly reduced sleep efficiency and increased waking after sleep onset. The reduction in sleep continuity may be due to the interrupted neuronal input to the cortex during sleep.

Brown et al. in a population based stroke surveillance study on 355 ischemic stroke patients concluded that ischemic stroke was not associated with the presence or severity of sleep discorded breathing [30].

### The stroke severity

In accordance with our study Cindy et al. studied 305 patients and showed that sleep apnea is common in the acute phase of ischemic stroke, and sleep apnea severity is associated with the higher NIHSS [31].

The study of the Bassetti and Aldrich [29] showed different results. It showed that the higher stroke severity was associated with shorter sleep time and lower sleep efficiency.The stroke scale used in this study was the Scandinavian Stroke Scale, not the NIHSS,which was an important difference, in addition to the smaller number of patients and early recording of polysomnography.

In the study of Terzoudi et al. [15] studied 62 acute stroke patients and 16 hospitalized controls an overnight Polysomnography was done. Sleep architecture was disturbed in stroke patients, where sleep stage one and REM sleep were negatively associated with stroke severity, and the latency to REM sleep is positively correlated with a good outcome. Sleep architecture is impaired in stroke patients, and this correlates with severity and outcome. Furthermore, the observation of similar REM sleep changes in patients with mild and severe stroke suggests that REM sleep abnormalities are more likely to reflect, in most cases, acute illness than brain damage^.^ REM sleep tends to be most affected early in the course of the illness and later it improves to recur to patient’s old pattern.

Contrary to our finding *Iddagoda *et al*.* studied 104 stroke patients in Western Australia and found that 29.8% post-stroke patients suffered from poor sleep. There was no relationship between poor sleep and the stroke characteristics, such as severity, side and type, or demographic characteristics. [32] this was also concluded by a study done by AK et al. they studied Sixty patients with a history of stroke aged under 55 years they all underwent Polysomnography and found No significant correlation between OSA severity and NIHSS [33].

In a Korean study done by Ahn et al. they prospectively studied 293 ischemic stroke patients and showed that SDB was very common in acute ischemic stroke. Also higher NIHSS score was associated with in the higher sleep disordered breathing (SDB) while the lesion location and specific stroke syndrome were not correlated with SDB [34].

## Conclusion

The microstructure of sleep is significantly impacted by cerebrovascular stroke. Brain stem strokes had the highest REM and REM latency, while cortical strokes had the highest moderate-to-severe AHI. Sizable strokes displayed increased indices of limb movement, desaturation, and oxygen saturation.

## Limitation

Due to difficulties in performing polysomnography safely, sizable brain stem strokes were omitted from the study.

## Data Availability

The research data supporting the results reported in the article is totally available upon request from the corresponding author.
